# Cytomegalovirus Pulmonary Involvement in an Immunocompetent Adult

**DOI:** 10.1155/2021/4226386

**Published:** 2021-08-10

**Authors:** Helena Luís, Carolina Barros, Mariana Gomes, José Luís Andrade, Nancy Faria

**Affiliations:** ^1^Department of Internal Medicine, Hospital Central do Funchal–SESARAM, E.P.E Avenida Luís de Camões Nº 57, Funchal 9004-514, Portugal; ^2^Department of Infectious Diseases, Hospital Central do Funchal–SESARAM, E.P.E Avenida Luís de Camões Nº 57, Funchal 9004-514, Portugal

## Abstract

**Introduction:**

Cytomegalovirus (CMV) is a linear double-stranded DNA virus that may cause severe and potentially fatal infection in immunocompromised hosts. In immunocompetent individuals, the infection is typically mild or asymptomatic. However, in the last years, some cases of severe cytomegalovirus infection in immunocompetent individuals have been described. *Clinical Presentation*. The authors present a male patient aged 42 years, without specific medical history, who presented a 15-day history of fever, headache, night sweats, odynophagia, and bilateral otalgia, without improvement after four days of therapy with amoxicillin/clavulanic acid. Blood count and biochemistry were performed with liver cytolysis pattern. Chest teleradiography showed diffuse interstitial infiltrate. Thoracic CT scan revealed areas in a ground glass with a cross-linking component in the left and right upper lung lobes compatible with an inflammatory/infectious process. Blood serology was positive for CMV IgG and IgM. The detection on blood and bronchoalveolar lavage of CMV DNA by polymerase chain reaction (PCR) was also positive. Ganciclovir was started based on the clinical features and the result of CMV serology. After 48 hours, there was a significant clinical improvement, with remission of fever, and he was discharged on the 13th day of hospitalization with oral valganciclovir, completing a 21-day antiviral course at home.

**Conclusion:**

With this clinical case, the authors highlight the importance of considering CMV infection in evaluating patients with pneumonia, even in immunocompetent ones, particularly in those with no clinical improvement with antibiotics instituted for bacterial pneumonia, and when other causes have been ruled out.

## 1. Introduction

Cytomegalovirus is a DNA virus belonging to the Herpesviridae family with a high serological prevalence in healthy adults (50 to 90%) [1], and in Portugal, it is about 77% [2]. It is transmitted by contact with infected body fluids or via the transplacental route [2, 3] and is often acquired in childhood or early adolescence [4].

The spectrum of diseases caused by CMV is diverse, depending fundamentally on the host. In immunocompromised hosts, infection by this agent causes significant morbidity and mortality, especially in transplant recipients and in those infected with the human immunodeficiency virus (HIV) [5, 6]. In the immunocompetent host, the infection is usually asymptomatic or can present as a mononucleosis-like syndrome [2, 4]. Some patients may experience prolonged fever for 2 to 3 weeks [2, 7]. Symptomatic infection involving the respiratory system appears to be rare (about 8%) [2, 8], presenting, usually, with respiratory failure and with interstitial infiltrate on chest X-ray [2]. The diagnosis is confirmed by serological testing (detection of CMV-specific immunoglobulin (Ig) M or a four-fold increase in CMV-specific IgG), molecular biology, and histological findings in lung biopsy [2].

Although ganciclovir or valganciclovir are currently recommended as first-line pharmacological agents in the treatment of immunocompromised patients with severe disease, there are still uncertainties regarding these agents' role in immunocompetent patients [4, 9].

## 2. Patient Information

A 42-year-old Caucasian male patient with no relevant medical history and no usual medication went to his attending physician for remitting fever (tympanic temperature (TT) maximum 38.9°C) associated with night sweats, odynophagia, and bilateral otalgia, with 15 days of evolution. He was medicated empirically with amoxicillin/clavulanic acid and paracetamol.

Four days later, he went to the emergency department (ER) due to the persistence of fever and the onset of nausea, asthenia, and intermittent occipital headache, intensity 8 out of 10 (numerical scale of pain), with irradiation to the frontal region, photo and sonophobia, and partial pain relief with paracetamol.

## 3. Clinical Findings

Upon observation, he was pale, had fever (TT 38°C), normotensive, and tachycardiac (115 bpm), with a respiratory frequency of 18 breaths/min and 98% of peripheral oxygen saturation. Cardiac and pulmonary auscultation did not reveal changes. The oropharynx had hyperemia and aphthous stomatitis. Otoscopy showed hyperemia of both tympanic membranes. Meningeal signs were negative.

## 4. Diagnostic Assessment

Performed blood count and biochemistry showed liver profile with liver cytolysis pattern –207 U/L aspartate aminotransferase (AST) and 125 U/L alanine aminotransferase (ALT); 692 U/L lactic dehydrogenase; normochromic anemia; ionogram and renal function without changes; C-reactive protein (CRP) 22.33 mg/L. No pulmonary or pleural involvement on chest X-ray. Cranial brain computed tomography (CT) showed no ischemic or hemorrhagic changes. He underwent a SARS-CoV-2 CRP test which was negative. We searched for hepatitis A antibody, hepatitis B surface antigen, and hepatitis C antibody, which were negative. Active infection by herpes simplex virus (HSV) 1 and 2 (IgM anti-HSV 1 and IgM anti-HSV 2 < 0.8), influenza A and B, respiratory syncytial virus (RSV), HIV 1 and 2, and Epstein–Barr (viral capsid antigen (VCA) IgG, VCA IgM, and Epstein–Barr nuclear antigen (EBNA) negatives) were also excluded. Enterovirus and parvovirus B19 infection were also excluded as well as leptospira infection. A therapeutic adjustment was performed with oseltamivir and ibuprofen.

Due to persistent fever and headache, he went to the ER again three days later. On admission, he reported dyspnea for medium exertion, intermittent chest pain, grade 6 out of 10, without irradiation and unrelated to body movements, and dry cough. On physical examination, he was subfebrile (TT 37.8°C) but hemodynamically stable. He maintained aphthous stomatitis and hyperemia of the oropharynx and tympanic membranes. Analytically, the maintenance of the pattern of liver cytolysis and elevation of CRP (46.52 mg/L) is highlighted (Table 1). Peripheral blood smear with 16% activated lymphocytes and normochromic normocytic anemia.

The patient was admitted to the Department of Internal Medicine, and the respective etiological study of the fever was carried out in the context of hospitalization. Blood and urine cultures were negative for bacteria and fungi. *Coxiella burnetii* and *Mycoplasma pneumoniae* antibodies' assays were negative. Urinary antigens for *Legionella* and *Streptococcus pneumoniae* were negative. Other serologies were negative: *Chlamydophila pneumoniae*, *Toxoplasma gondii*, and VDRL. The lumbar puncture excluded infection of the central nervous system. We searched for autoimmune diseases (lupus anticoagulant, anticardiolipin antibodies, antinuclear antibodies, anti-smooth muscle antibodies, and anti-double-strand DNA antibodies) but were negative.

**Figure 1:**
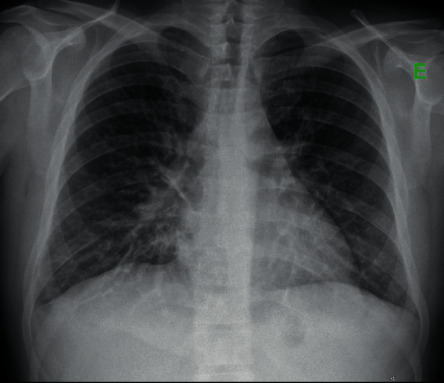
Chest X-ray performed on the 2^nd^ trip to the ER: bilateral perihilar reinforcement, erasure of both costophrenic sinuses, and diffuse interstitial infiltrate are observed.

**Figure 2:**
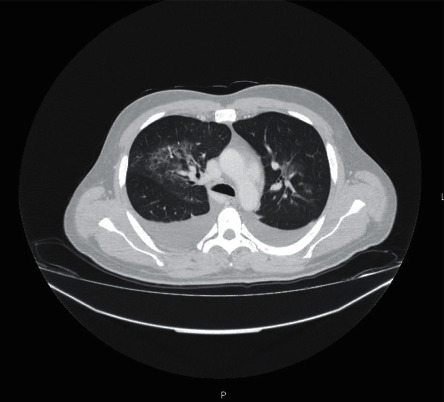
Chest CT with ground glass areas in the left and right pulmonary lobes; bilateral pleural effusion.

From the imaging study carried out, bilateral perihilar reinforcement, erasure of the costophrenic sinuses, and diffuse interstitial infiltrate were found in chest teleradiography (Figure 1). Thoracic-abdominal-pelvic CT showed areas in a ground glass with a cross-linking component in the left and right upper lung lobes compatible with an inflammatory/infectious process (Figure 2). It also revealed the presence of bilateral pleural effusion, hepatomegaly with periportal and perihepatic edema, splenomegaly, and free fluid in a right paritetocolic drip. A transthoracic echocardiogram was performed showing no abnormal changes.

Initially, since the patient was considered immunocompetent, CMV serology was not requested. However, and after excluding all possible etiologies, they were researched. Alterations compatible with CMV primoinfection were detected—anti-CMV IgM antibodies 6.91 S/CO and anti-CMV IgG 21.4 UA/mL. The detection on blood of CMV DNA by real-time quantitative polymerase chain reaction (PCR) was positive with 91880 copies/mL. Bronchoscopy did not reveal bronchial abnormalities. Bronchoalveolar lavage (BAL) cytology was negative for viral inclusions, and no organisms were isolated from cultures. CMV real-time polymerase chain reaction (PCR) on BAL was positive (704 copies/mL).

## 5. Therapeutic Intervention

Acute CMV pneumonia in an immunocompetent host was assumed, and antiviral therapy was started with ganciclovir at a dose of 375 mg (5 mg/kg, patient weight 75 kg) intravenously at 12/12 h. We chose for ganciclovir based on the clinical features and the result of CMV serology. We performed a dilated eye exam which did not show any ocular damage. After 48 hours, there was a significant clinical improvement, with remission of fever. He was discharged on the 13^th^ day of hospitalization after finishing four days of ganciclovir. Since we do not have oral formula of ganciclovir in our country, the patient was medicated with oral valganciclovir 900 mg every 12 hours for 21 days, after which complete resolution of symptoms was noted.

## 6. Follow-Up and Outcomes

**Figure 3:**
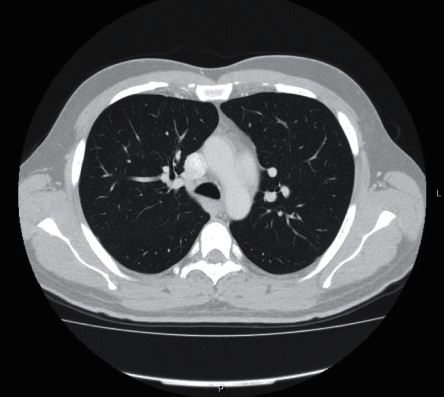
Follow-up chest CT showing resolution of the ground glass areas and the bilateral pleural effusion.

Thoracic-abdominal-pelvic CT, performed three months after discharge, revealed resolution of the initial findings (Figure 3). Currently, the patient is healthy and without the need for therapy.

## 7. Discussion

The authors conducted a literature search, through the PubMed platform, of articles on CMV infection in immunocompetent individuals, using the words: cytomegalovirus, pneumonia, and immunocompetent, until January 20, 2021. They collected a total of 17 cases of CMV pneumonia (Table 2).

The authors found that all cases occurred in developed countries, which may reflect underdiagnosis in developing countries. Ages range from 10 to 73 years, with an average of 40 years. Of the 17 cases, 11 patients were found to be male and 6 were females.

To date, few cases of prolonged symptomatic infection have been described, with the majority of studies reporting cases of hepatitis and colitis [9]. In a retrospective study, carried out by Faucher et al., in a total of 116 patients hospitalized and followed up on an outpatient basis, it was found that 99% had a fever with an average duration of 21 days [25]. Headache was described by 51%, while 46% had myalgia and 36% had splenomegaly [25]. Pulmonary symptoms were described in 8% of patients and neurological symptoms in 1% [25]. Pulmonary involvement of CMV disease is rare. Patients may present a dry cough or develop severe interstitial pneumonia, making it necessary to perform a differential diagnosis with other viral infections as influenza or adenovirus [2]. Although hypoxemia is frequently present [2], our patient did not develop in the disease course.

Regarding signs and symptoms, most of the patients studied had fever, malaise, flu-like syndrome, dyspnea, and cough. Some patients had involvement of other organ systems with jaundice and abdominal pain. Only 2 of the 17 patients had associated comorbidities such as atrial fibrillation, arterial hypertension, and obstructive sleep apnea syndrome. Only one patient was a smoker.

Since there is no specific radiological pattern of pulmonary involvement, the patient may present with an absent or minimal initial pulmonary infiltrate or with diffuse interstitial infiltrates [2]. Most of the patients analyzed had diffuse interstitial infiltrates on chest teleradiography and only one patient with localized disease, described by Gonçalves et al.

Laboratory diagnosis can be performed through serological tests or changes in biochemistry. Serology is based on the elevation of the CMV IgM antibody titer or an increase in the IgG antibody titer. In the clinical cases studied, the diagnosis of CMV pneumonia was made by serology established on IgM antibodies, cultures, or histology. In patients who died, the diagnosis was established from the findings of the histological examination during the autopsy.

In the clinical case presented, the diagnosis of infection by CMV was suspected after the exclusion of other etiologies and confirmed by the elevated titers of IgM and IgG CMV and by the detection of CMV DNA by PCR.

According to the Centers for Disease Control and Prevention, there is no treatment established for CMV infection in immunocompetent patients [2]. The literature on current practices, although limited, suggests that antiviral therapy directed with ganciclovir or valganciclovir is suitable for severe infection in immunocompetent individuals [2]. Ganciclovir was patented in 1980 and approved for the treatment of CMV infection in 1988 [24]. Valganciclovir was approved for medical use in 2001. Seven of the patients had CMV pneumonia before ganciclovir was approved, in which five of them died, and two survived. After the approval of ganciclovir, all the patients who received antiviral therapy survived. In the cases where authors described the administered dose, most of the patients received 900 mg of valganciclovir twice a day. The duration of treatment ranged from 4 to 21 days despite significant clinical improvement in the first days. In this presented patient, as soon as he started antiviral therapy with ganciclovir, there was a significant clinical improvement. All patients who were treated with ganciclovir or valganciclovir showed good results and none of them reported long-term sequelae.

With this clinical case, the authors highlight the importance of considering CMV infection in evaluating patients with pneumonia, even in immunocompetent ones, particularly in those with no clinical improvement with antibiotics instituted for bacterial pneumonia, and when other causes have been ruled out.

## Figures and Tables

**Table 1:** Analytical evolution from the 1^st^ visit to ER up to 8 days of therapy with valganciclovir.

	1^st^ trip to ER	2^nd^ trip to ER	2^nd^ day of hospitalization	9^th^ day of hospitalization	13^th^ day of hospitalization	8^th^ day on valganciclovir
Hemoglobin (g/dL)	11.1	9.7	8.9	8.9	11,5	12
VCM (fL)	86.4	88.3	88.3	90.3	91,1	90,4
HCM (pg)	29.8	30.1	29.3	29.5	29,4	31,2
Prothrombin time (seg)	17.6	15.8	15.4	—	14,3	12,7
INR (ratio)	1.5	1.3	1.3	—	1,2	1,1
ALT/GPT (U/L)	199	153	249.9	190.6	120,4	68,8
AST/GOT (U/L)	207	95.9	192.2	79	56,4	35,4
GGT (U/L)	—	—	197.3	381.8	291,2	164,0
LDH (U/L)	692.0	749.0	885.	792	631,0	323,0
TBIL (mg/dL)	—	0.9	0.8	0.7	0,7	0.5
ALP (U/L)	—	—	106.	143	121,0	105,0
CRP (mg/L)	22.3	46.5	55.4	26	6,8	—
Anti-CMV IgG (UA/mL)	—	—	21.4	—	75,7	71,3
Anti-CMV IgM (S/CO)	—	—	6.9	—	9,0	6,5
DNA (PCR) – cytomegalovirus (copies/mL)	—	—	91880	—	<250	<250

**Table 2:** Published clinical cases of CMV pneumonia in immunocompetent individuals.

Authors	Year	Country	Age/sex	Diagnostic method	Antiviral therapy	Outcome
Vogel [10]	1958	United States of America	10/M	Histology	None	Died
Shoyama et al. [11]	1969	Japan	63/F	Histology	None	Died
Ii K et al. [12]	1972	Japan	73/M	Histology	None	Died
Bäck et al. [13]	1977	Scandinavia	53/M	Serology	None	Survived
Lipton et al. [14]	1981	United States of America	28/F	Serology, histology, immunofluorescence	None	Died
Teixidor et al. [15]	1982	United States of America	21/F	Histology	None	Died
Cohen et al. [16]	1985	United States of America	34/M	Serology	None	Survived
Manian and smith [17]	1993	Sweden	32/F	Serology, culture	Ganciclovir	Survived
Halimi et al. [18]	1993	France	40/F	Serology, culture, immunohistochemistry for CMV antigen in bronchoalveolar lavage	Ganciclovir	Survived
McCormak et al. [19]	1998	Australia	31/M	Serology	Ganciclovir	Survived
Karakelides et al. [20]	2003	United States of America	47/M	Histology	None	Survived
Cunha et al. [21]	2008	United States of America	64/M	Serology	None	Survived
Barclay et at. [22]	2011	UK	38/F	Serology, PCR on blood	Valganciclovir	Survived
Grilli et al. [8]	2012	Italy	29/M	Serology	Valganciclovir	Survived
Müller et al. [23]	2016	Germany	49/M	Serology	Valganciclovir	Survived
Gonçalves et al. [2]	2018	Portugal	29/M	Serology	Valganciclovir	Survived
Waqas et al. [24]	2019	United States of America	38/M	Serology, culture	Valganciclovir	Survived

## Data Availability

The data used to support the findings of this study are available from the corresponding author upon request.
